# Deposition of Visible Light Active Photocatalytic Bismuth Molybdate Thin Films by Reactive Magnetron Sputtering

**DOI:** 10.3390/ma9020067

**Published:** 2016-01-22

**Authors:** Marina Ratova, Peter J. Kelly, Glen T. West, Xiaohong Xia, Yun Gao

**Affiliations:** 1 Surface Engineering Group, School of Engineering, Manchester Metropolitan University, Manchester M1 5GD, UK; peter.kelly@mmu.ac.uk (P.J.K.); g.west@mmu.ac.uk (G.T.W.); 2 Hubei Collaborative Innovation Center for Advanced Organic Chemical Materials, Ministry-of-Education Key Laboratory for the Green Preparation and Application of Functional Materials, Faculty of Materials Science and Engineering, Hubei University, Wuhan 430062, China; xhxia@hubu.edu.cn (X.X.); gaoyun@hubu.edu.cn (Y.G.)

**Keywords:** bismuth molybdate, photocatalytic coatings, magnetron sputtering, visible light, thin films

## Abstract

Bismuth molybdate thin films were deposited by reactive magnetron co-sputtering from two metallic targets in an argon/oxygen atmosphere, reportedly for the first time. Energy dispersive X-ray spectroscopy (EDX) analysis showed that the ratio of bismuth to molybdenum in the coatings can be effectively controlled by varying the power applied to each target. Deposited coatings were annealed in air at 673 K for 30 min. The crystalline structure was assessed by means of Raman spectroscopy and X-ray diffraction (XRD). Oxidation state information was obtained by X-ray photoelectron spectroscopy (XPS). Photodegradation of organic dyes methylene blue and rhodamine B was used for evaluation of the photocatalytic properties of the coatings under a visible light source. The photocatalytic properties of the deposited coatings were then compared to a sample of commercial titanium dioxide-based photocatalytic product. The repeatability of the dye degradation reactions and photocatalytic coating reusability are discussed. It was found that coatings with a Bi:Mo ratio of approximately 2:1 exhibited the highest photocatalytic activity of the coatings studied; its efficacy in dye photodegradation significantly outperformed a sample of commercial photocatalytic coating.

## 1. Introduction

Semiconductor photocatalysis is gradually becoming a popular method of surface depollution and decontamination in the 21st century. Since the pioneering publication of Fujishima and Honda in 1969 [1], the number of publications describing photocatalytic materials increases annually. Practical applications of photocatalysts range from water splitting to the degradation of organic pollutants and surface disinfection [2,3,4,5,6]. Although titanium dioxide, as the first studied and most widely available photocatalyst, remains the key material in semiconductor photocatalysis [7], the number of publications describing the use of alternative photocatalytic materials is increasing rapidly. This is due to the fact that, being a wide band gap semiconductor, titanium dioxide can be activated only with UV irradiation (4% of the solar spectrum, compared to 43% for visible light [8]), which makes its use inefficient for many practical applications, particularly where an extra UV light is not available. The low quantum efficiency of titanium dioxide remains another limiting factor to the development of efficient photocatalytic materials based on TiO_2_; conventional methods, such as doping or photosensitization, only partly solve this issue. Therefore, improvements in photocatalytic efficiency, along with the introduction of visible light activity, remain important challenges. Since no method of fully solving the issues of titanium dioxide has been proposed to date, there is an obvious need for the development of efficient visible light activated low band gap semiconductors, thus providing an effective, environmentally friendly solution for air and water decontamination processes. Zinc oxide and cadmium sulphide are the materials most often used as alternatives to titanium dioxide [9], however, zinc oxide, similar to TiO_2_, is characterized with a wide band gap (3.0 eV) and demonstrates an instability in UV-irradiated aqueous solutions, while although CdS is a narrow band gap semiconductor, it has toxicity issues that significantly limit its use.

Bismuth complex oxides are often referred to as examples of narrow band gap visible-light activated and efficient photocatalytic materials, with bismuth molybdate and bismuth tungstate being the most studied members of the bismuth complex oxides family. Bismuth molybdate is an Aurivillius oxide with formula Bi_2_MoO_6_; it is well described in the literature due to its interesting physical and chemical properties. It finds applications as a gas sensor [10], dielectric, and has recently been reported as an efficient visible light-activated photocatalyst [11]. The interest in bismuth-based photocatalysts arises due to the fact that they are typically characterized with low band gap values, as the result of hybridization between the Bi 2*s* and O 2*p* states [12]. Thus, in various studies bismuth molybdate photocatalysts were successfully used for the degradation of such dyes as methylene blue [13], rhodamine B [14,15,16], indigo carmine [17], the removal of antibiotics from the wastewater [8], and the decomposition of phenol [15]. Photocatalytic bismuth molybdates are typically synthesized using chemical methods, such as the hydrothermal method [8,18,19], molten salt method [20], solid state reaction [17] or the Pechini method [21]. However, to the best of our knowledge there are no commercial photocatalytic products based on the use of bismuth complex oxides available at the present time. Furthermore, very few data are available on the photocatalytic behavior of thin films of bismuth molybdates. Physical deposition methods, and magnetron sputtering in particular, offer significant advantages for the deposition of thin films onto a variety of substrates [22]. The magnetron sputtering technique is a versatile, scalable method that allows control of such parameters as crystallinity, composition, and thickness. Literature searches revealed several works where authors used magnetron sputtering method for deposition of bismuth complex oxides, however all these publications describe the RF sputtering of a compound oxide target, rather than the reactive sputtering technique from metallic targets [23]. The present work introduces the preparation of thin bismuth molybdate coatings using reactive magnetron sputtering, reportedly for the first time. The authors of the present work recently demonstrated that reactive magnetron sputtering could be successfully applied for the deposition of bismuth tungstate, which also exhibits photocatalytic properties under visible light irradiation [24]. Similar to bismuth tungstate, bismuth molybdate coatings can be deposited by the reactive co-sputtering of two metallic targets and used as an efficient visible light-activated photocatalyst for the degradation of organic dyes. This paper describes the deposition, characterization and testing of bismuth molybdate coatings produced via this technique and demonstrates that, unlike a commercial titania-based photocatalytic product, the Bi_2_MoO_6_ coatings can strongly degrade organic dyes under visible light irradiation.

## 2. Materials and Methods

### 2.1. Deposition of the Coatings

**Figure 1:**
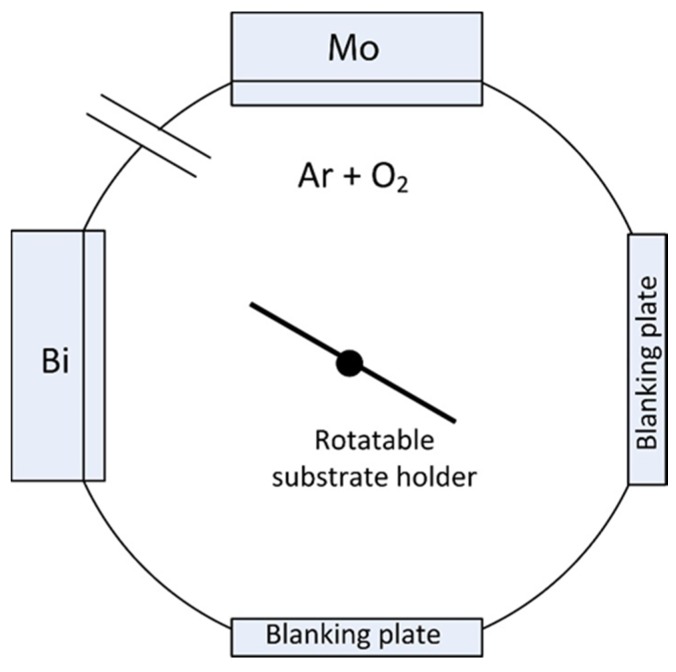
Schematic representation of Teer UDP450 sputtering rig.

Bismuth molybdate coatings were deposited in a Teer UDP450 sputtering rig (Teer Coatings Ltd., Droitwich, UK) from two 300 mm × 100 mm vertically opposed type II unbalanced planar magnetrons, installed through the chamber walls in a closed field configuration. The arrangement was similar to the one reported in earlier work [24], with a bismuth target fitted on one of the magnetrons and molybdenum target on the other one; both targets used were 99.95% purity. The schematic representation of the Teer UDP450 sputtering rig is given in the Figure 1. The targets were driven in pulsed DC mode using a dual channel Advanced Energy Pinnacle Plus power supply (Advanced Energy, Fort Collins, CO, USA); a pulse frequency of 100 kHz and duty of 50% (synchronous mode) were used for all the deposition runs. The target powers were varied systematically in the range from 100 to 500 W to vary the bismuth:molybdenum ratio in the coatings. The reactive sputtering process was carried out in an argon/oxygen atmosphere at a pressure of 0.3 Pa. The gas flows were controlled with mass flow controllers: 40 sccm of argon and 28 sccm of oxygen. The soda-lime glass substrates used in this work were ultrasonically cleaned in propanol prior to deposition and mounted on the electrically floating rotatable substrate holder. The rotation speed was set at 8 rpm for all coatings produced in this work. The distance between targets and substrate holder was 10 cm.

### 2.2. Characterization of the Coatings

The composition of the coatings was investigated with EDX (EDAX Trident, installed on a Zeiss Supra 40 VP-FEG-SEM, Edax Co., Mahwah, NJ, USA). Crystallographic phases of the coatings were analysed with Raman spectroscopy (Renishaw Invia, 514 nm laser) (Renishaw, Gloucestershire, UK) and X-ray diffraction (Panalytical Xpert powder with CuKa1 radiation at 0.154 nm in grazing incidence mode at 3°) (PANalytical Ltd., Cambridge, UK). Coating thickness was measured using a Dektak^TM^ surface profilometer (Bruker, Billerica, MA, USA). Surface roughness of the coatings was determined using a Zemetrics 3D optical profilometer (Zygo Corporation, Middlefield, CT, USA). The oxidation state information was obtained by X-ray photoelectron spectroscopy (XPS) (Thermo Scientific™ ESCALAB™ 250Xi employing AlKα source with the pass energy of 20 eV, Waltham, MA, USA). Finally, values of the optical band gaps of the coatings were calculated using the Tauc plot method [25], by plotting (αhν)^1/2^ as a function of hν and extrapolating the linear region to the abscissa (where α is the absorbance coefficient, h is Plank’s constant, ν is the frequency of vibration). Transmittance data, used for band gap calculation, were determined using an Ocean Optics USB 2000+ spectrometer (Ocean Optics Inc., Oxford, UK).

### 2.3. Photocatalytic Activity Assessment

**Figure 2:**
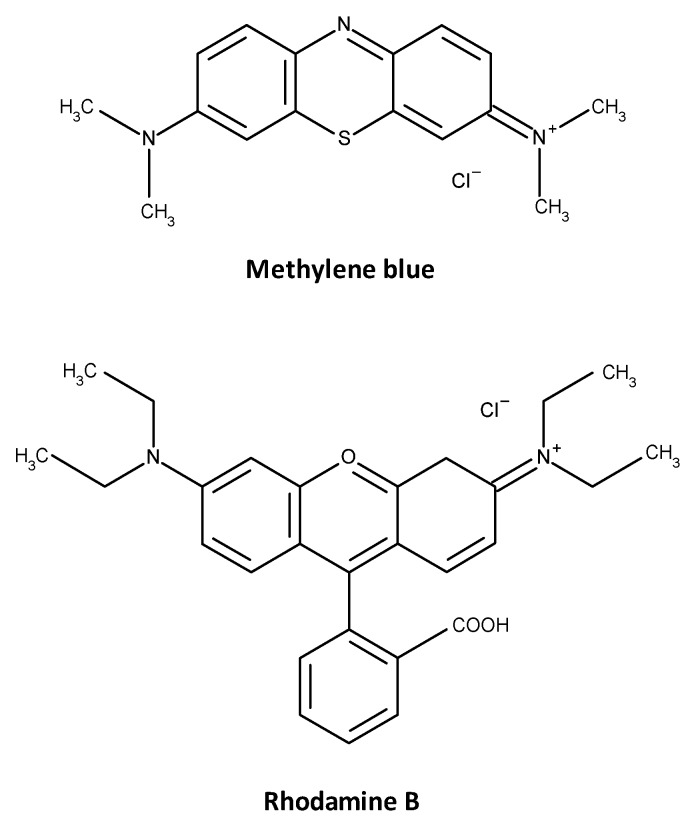
Molecular structures of methylene blue and rhodamine B.

As degradation of the organic compounds is one of the main application of photocatalytic materials, the photocatalytic activity of the bismuth molybdate coatings was assessed using the decomposition of two model pollutant dyes, namely methylene blue (MB) and rhodamine B (RhB) (all reagents were purchased from Sigma Aldrich, St. Louis, MO, USA). The set of two dyes was used to improve reliability of the test performed, as there are certain limitations associated with the use of dye degradation test under the visible light source [26]. The chemical structures of methylene blue and rhodamine B are shown in Figure 2. Methylene blue is an organic dye with chemical formula C_16_H_18_ClN_3_S. Aqueous solutions of methylene blue have an intense blue color, with an absorbance peak at 665 nm.

Degradation of methylene blue is frequently used as a measure of photocatalytic activity. Rhodamine B is an organic dye with chemical formula C_28_H_31_ClN_2_O_3_; aqueous solutions of RhB are characterized with an intense pink color, and consequently have an absorbance peak at 554 nm.

Prior to the photocatalytic measurements, the specimens (1.5 × 2.5 cm^2^) were immersed in a conditioning solution of dyes for a total time of 30 min in the dark to reach adsorption–desorption equilibrium. The photodegradation of the dyes was measured based on the absorbance peak height decay rate. The testing solution concentrations were 1.5 µmol/L for MB and 20 µmol/L for RhB; both concentrations were defined experimentally prior to the photocatalytic experiment to be able to detect photocatalytic responses of each coating in 1 h experiments. Samples were immersed into 40 mL of dye solution with continuous magnetic stirring, and the absorbance peak heights were monitored in continuous mode with an Ocean Optics USB 2000+ spectrometer for a total time of 1 h. According to the Lambert-Beer law, the concentration of the dye is proportional to the absorbance decay. Photocatalytic dye decomposition was approximated to pseudo-first order kinetics:

(1) $$\frac{\ln C_{0}}{C}=-k_{a}t$$

where *C*_0_ and *C* are concentrations of the test dye before the experiment and after 1h experimentation, respectively; *k*_a_ is the first order reaction constant; *t* is the experiment time. Therefore, the first order reaction constants were determined as the gradients of the plots ln(*A*_0_/*A*) *versus* time (where *A*_0_ and *A* are the peak absorbance values of the test dye before the experiment and after 1 h, respectively).

The visible light source used at this work was simulated by combining a fluorescent light source (2 × 15 W Ushio fluorescent lamps (USHIO, Tokyo, Japan)) with a 395 nm long pass filter (Knight Optical, Harrietsham, UK) to filter off the UV component of the fluorescent light source. The emission spectrum of the light source is given elsewhere [27]. A series of reference tests was carried out prior to the photocatalytic activity measurements, including testing under visible light with a blank sample (soda-lime glass) and testing each sample in dark conditions, to prove that solution decolorization was caused by photodecomposition of the dyes, rather than any side reactions. The reference tests showed that when either of the two dyes were tested with no photocatalyst present, the absorbance peak height decay in a 1 h experiment was not more than 1%, therefore this effect could be neglected in further calculations of the photocatalytic activity. Photocatalytic activity values obtained for the bismuth molybdate coatings deposited for this work were compared to the photocatalytic performance of commercial photocatalytic coatings of identical geometrical size (Pilkington Activ^®^ glass, NSG UK, Ormskirk, UK).

## 3. Results and Discussion

### 3.1. Coatings Overview

An overview of the coating deposition conditions, composition, thickness (measured by stylus profilometry and verified by optical profilometry), and appearance is presented in Table 1.

**Table 1:** Compositional properties and thickness of bismuth molybdate coatings.

Sample ID	Power on Bi Target (W)	Power on Mo Target (W)	Content of Bi (at.%)	Content of Mo (at.%)	Coating Thickness (nm)	Coating Visual Appearance	Average Transmittance Value in the Visible Part of the Spectrum (%)
BMO1	100	500	46	54	180	Light yellow, transparent	79.7
BMO2	125	500	52	47	185	Light yellow, transparent	80.2
BMO3	150	450	64	36	200	Light yellow to light brown, transparent	74.2
BMO4	200	400	69	31	310	Light brown, transparent	70.4
BMO5	300	300	78	22	350	Light brown, transparent	70.1

As can be seen from the data presented in the table, varying the power to the bismuth and molybdenum targets allowed the deposition of coatings with different Bi:Mo ratios. In terms of visual appearance, all the coatings produced were optically transparent and uniform, with no visual signs of stresses. The color of the coatings varied, depending on the bismuth:molybdenum ratio, from light yellow for the coatings with higher bismuth content to light brown for samples BMO4 and BMO5. It is clear from the table that increasing the power to the bismuth target resulted in the deposition of thicker coatings, as bismuth has a significantly higher sputtering yield than molybdenum. It has been reported elsewhere that this is due to polyatomic sputtering [28]. This point can also be observed from the composition data. For example, coating BMO2 has a Bi:Mo atomic ratio close to unity, but the power to the Mo target was 4 times greater than the power to the Bi target. Additionally, when the target powers were equal (coating BMO5), the Bi:Mo atomic ratio was almost 4:1.

Based on the XRD and Raman spectroscopy results, all the as-deposited coatings were characterized with amorphous or weakly crystalline microstructures. They were, therefore, annealed in air at 673 K for 30 min and then allowed to cool in air gradually for 5–6 h to avoid the formation of thermal stresses in the coatings.

### 3.2. Raman Spectroscopy Results

**Figure 3:**
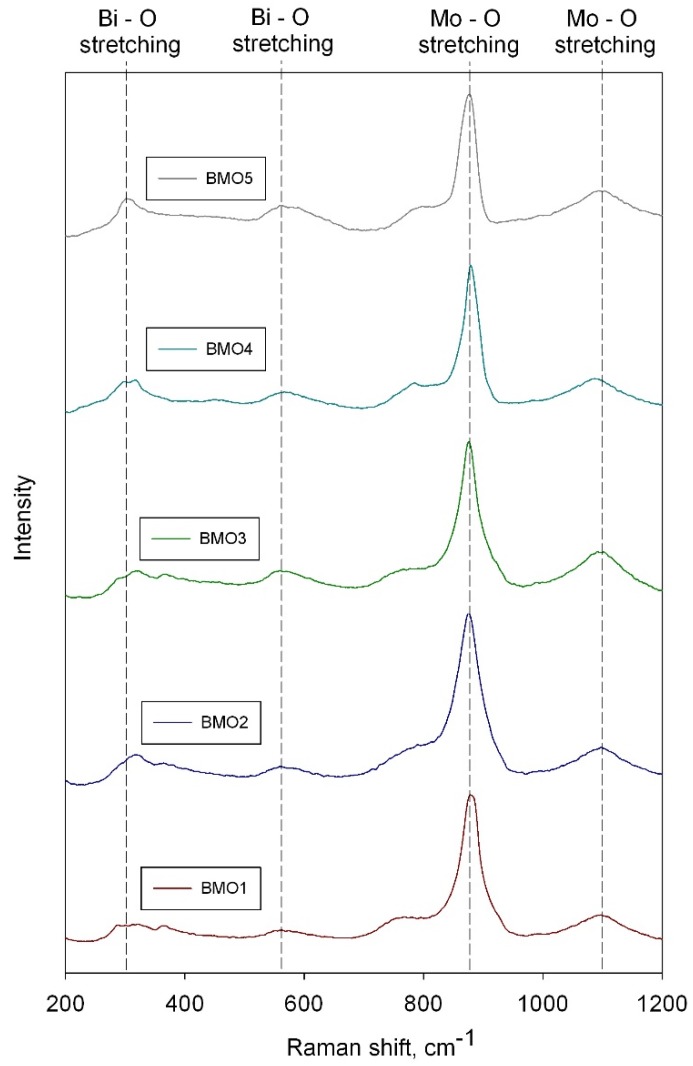
Results of Raman spectroscopy for bismuth molybdate coatings deposited onto glass substrates and annealed at 673 K.

Raman spectroscopy is a powerful technique for phase characterization of transition metal oxides by recording and assigning different frequencies of Raman spectra to different molecular structures. The Raman spectra of the coatings annealed at 673 K are shown in the Figure 3. It can be seen that all the samples showed characteristic bands of γ-Bi_2_MoO_6_, with very strong bands at around 880 cm^−1^ and medium bands at around 1100 cm^−1^ which correspond to Mo–O bond stretching, and medium bands in the region of 570 and 315 cm^−1^, corresponding to Bi–O–Mo and/or Bi–O bonds stretching [29,30]. This information is in good agreement with Raman spectra literature data for thermally treated γ-Bi_2_MoO_6_ [31].

### 3.3. XRD Results

**Figure 4:**
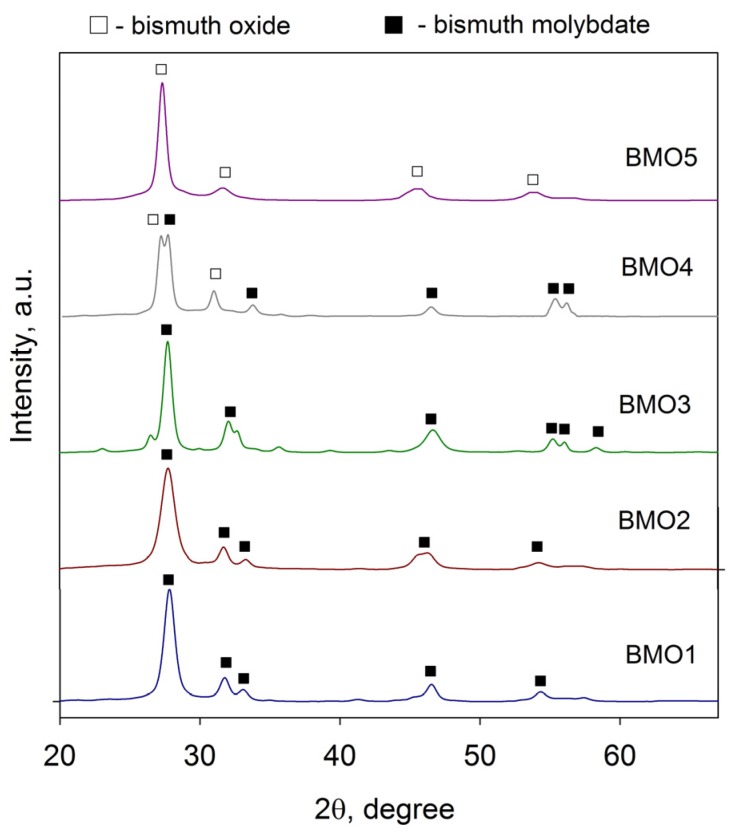
XRD patterns of bismuth molybdate coatings deposited onto glass substrates and annealed at 673 K.

The XRD results, presented in Figure 4, confirm that after thermal treatment all the coatings were crystalline. The peaks observed on the XRD patterns of samples BMO1–BMO4 correspond to γ-bismuth molybdate (JCPDS: 22-0113). Several additional peaks can be clearly seen on the XRD pattern of the coating BMO4 that can be attributed to bismuth oxide (JCPDS: 16-0654). Unlike the other coatings, sample BMO5 exhibited only a bismuth oxide crystal phase (JCPDS: 16-0654), with characteristic diffraction peaks at 27.3° 2θ. It is obvious that bismuth oxide peaks appear as the bismuth content in the coatings increases. It should be noted that no additional molybdenum oxide peaks were found on any of the diffraction patterns.

### 3.4. XPS Results

**Figure 5:**
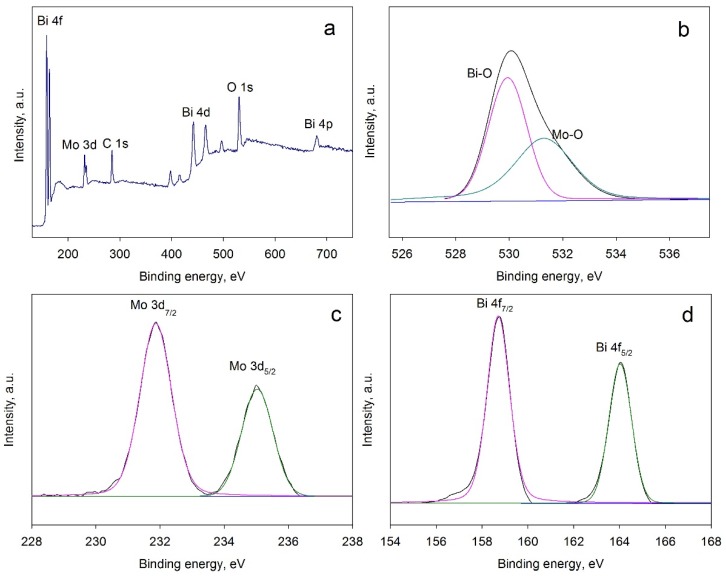
XPS results for coating BMO3: (**a**) Survey spectrum; (**b**) O 1s spectrum; (**c**) Mo 3d spectrum; (**d**) Bi 4f spectrum.

The XPS results for coating BMO3 are presented in Figure 5.

The survey spectrum is represented in Figure 5a; the presence of the carbon (C 1s peak) is typically explained by adventitious carbon contamination. Judging from the position of this peak, which is normally observed at 284.8 eV and commonly used as reference peak [32], it can be seen that the peaks are shifted to lower binding energies by approximately 2.5 eV, with the C 1s peak at 282.3 eV in the present study. Peak shifts are often reported and typically explained by positive charge build up on the sample [33]. The high resolution XPS of O 1s is shown in Figure 4b. The peak is mainly attributed to Bi–O bonds at 527.4 eV and to Mo–O bonds at 528.8 eV [34]. High resolution Mo 3d and Bi 4d peaks are represented in Figure 5c,d, respectively. The peaks at 229.4 and 232.5 eV (Figure 5c) can be attributed to the Mo^6+^ oxidation state, while the peaks at 156.2 and 161.5 eV (Figure 5d) are assigned to the Bi^3+^ oxidation state.

### 3.5. Band Gap Calculation

**Figure 6:**
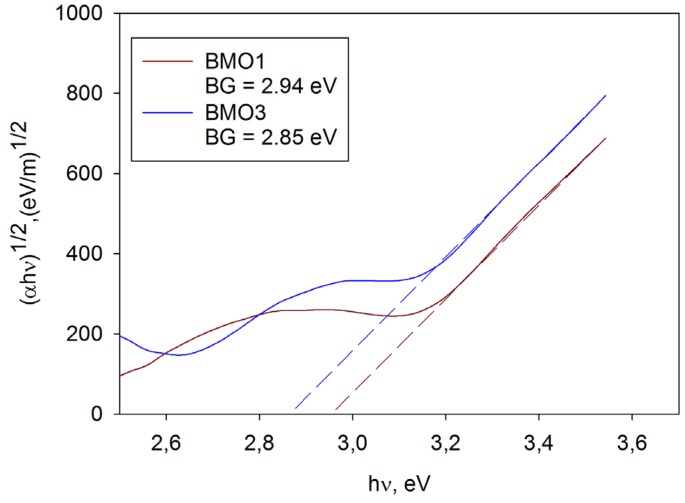
Examples of band gap calculation for coatings BMO1 and BMO3.

Optical band gap values of the coatings were estimated using the classical Tauc plot method for crystalline semiconductors, as described earlier in the Experimental section. Examples of band calculation for coatings BMO1 and BMO3 are shown in Figure 6. The calculated values of the optical band gaps for all studied coatings are given in the Table 2.

It can be seen that the bad gap data are in good agreement with earlier studies of photocatalytic bismuth molybdates, where the band gap values typically range from 2.56 to 3 eV [16,35]. However, it should be noted that all the band gap data are estimated values only, as a variety of bismuth molybdate phases with different stoichiometric ratios may be formed as a result of annealing. From the values of the band gaps calculated it can be concluded that the deposited coatings are expected to be activated by light of wavelengths below 421 nm (for sample BMO1) to 468 nm (for sample BMO5), therefore all the photocatalytic coatings studied could be activated with the light source used in this work. It is obvious that higher molybdenum contents in the coatings blue shifted the band gap, while applying higher power to the bismuth target resulted in a shift of the band gap towards the visible range.

**Table 2:** Band gap and surface roughness values and dye degradation rate constants for bismuth molybdate coatings.

Sample ID	Predominant Crystal Phase	Band Gap (eV)	Surface Roughness *S*_a_ (nm)	Methylene Blue *k*_a_ × 10^−5^ (s^−1^)	Rhodamine B *k*_a_ × 10^−5^ (s^−1^)
BMO1	Bismuth molybdate	2.94	5.3	0.8	0.6
BMO2	Bismuth molybdate	2.94	5.6	1.2	1.0
BMO3	Bismuth molybdate	2.85	5.1	2.0	2.6
BMO4	Bismuth molybdate/bismuth oxide	2.74	5.6	1.1	1.3
BMO5	Bismuth oxide	2.65	5.7	0.4	0.5

### 3.6. Surface Roughness

The surface roughness of the coatings was evaluated by means of white light surface profilometry, and S_a_ values for the coatings are given in the Table 2, which indicates that all the coatings were of similar surface roughness. Thus, their photocatalytic activity values can be compared directly, with differences attributed to variations in composition, crystallinity and optical properties, rather than to variation in contact area with the pollutants used for photocatalytic activity evaluation. A sample of commercial coating used for comparison purposes was characterized with having a considerably higher surface roughness value (*S*_a_ = 12.6 nm), therefore providing higher contact area with the dyes used as model pollutants.

### 3.7. Photocatalytic Activity

**Figure 7:**
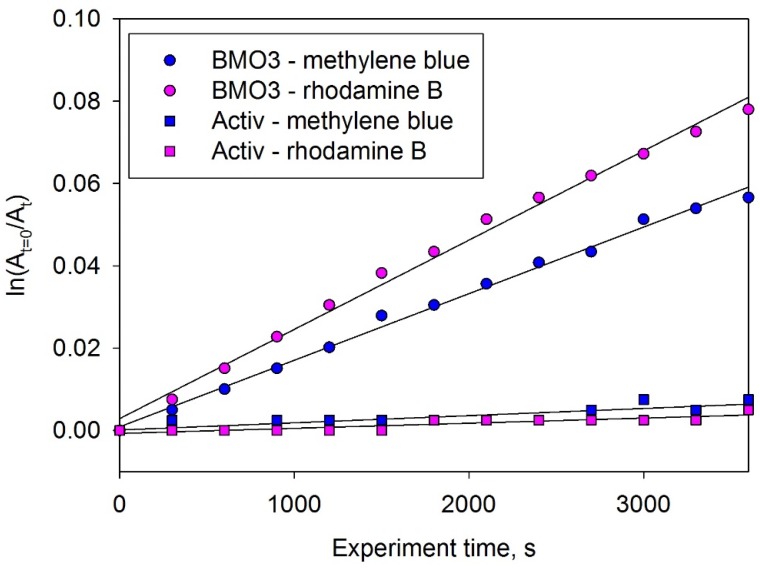
Degradation kinetics of in contact with sample BMO3 and Activ^®^ under visible light irradiation.

The photocatalytic activities of bismuth molybdate coatings were evaluated by monitoring the decomposition of methylene blue and rhodamine B organic dyes under visible light irradiation. Control experiments showed that neither of the chosen model pollutants were degraded by bismuth molybdate coatings in the dark or being illuminated by visible light in the absence of the photocatalyst. Examples of the dye degradation kinetics of methylene blue and rhodamine B for coating BMO3 are shown in Figure 7. Corresponding decay rates for a sample of commercial photocatalytic material (Pilkington Activ^®^ glass) of identical geometrical size are given for reference purposes. Despite a higher value of surface roughness, being a titania-based photocatalytic coating, Activ^®^ glass not surprisingly demonstrated very low photocatalytic activity under visible light irradiation (the first order rate constants for degradation of methylene blue and rhodamine B were 0.1 × 10^−5^ and 0.2 × 10^−5^ s^−1^, respectively). The values of first order rate constants of dye degradation reaction under visible light for the studied bismuth molybdate coatings are given in Table 2 for a quantitative characterization of the photodegradation process. The photocatalytic activity of all the deposited coatings was superior to that determined for the commercial photocatalyst. Of the coatings studied, it can be seen that sample BMO3 demonstrated the highest photocatalytic efficacy against either of two dyes used. From the EDX results, the ratio of Bi:Mo in this coating was 64:36, which is close to the stoichiometric composition of bismuth molybdate Bi_2_MoO_6_.

In a similar manner to magnetron sputtered bismuth tungstate coatings studied earlier [24], it was found that increasing the bismuth content in the coatings lowered the photocatalytic efficiency, despite reducing the band gap value. As only diffraction peaks for bismuth oxide could be seen on the XRD pattern of coating BMO5, this suggests that the lower photocatalytic activity of the coatings with higher bismuth content can be attributed to the low photocatalytic activity of bismuth oxide, as very few publication refer to Bi_2_O_3_ as a photocatalytic material [36,37]. In contrast, coatings with higher molybdenum content exhibited significantly higher values of the band gap, therefore their low photocatalytic activity under visible light can be explained by the smaller portion of the visible spectrum available for their activation.

### 3.8. Photocatalyst Reusability Evaluation

**Figure 8:**
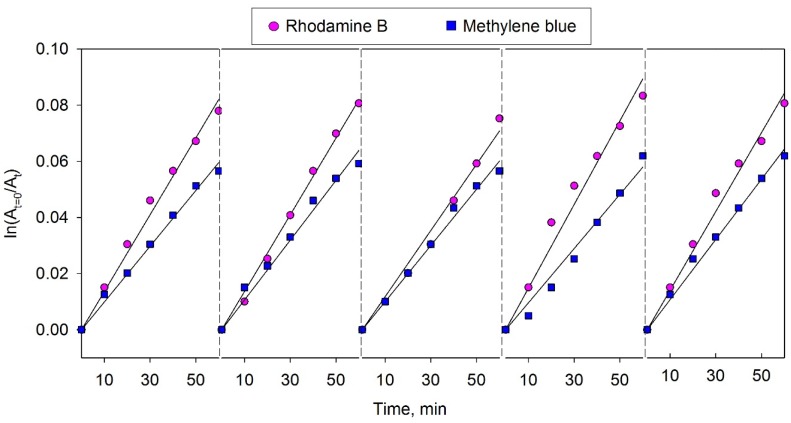
Degradation kinetics of MB and RhB during 5 cycles of dye photodegradation using sample BMO3 under visible light irradiation.

It is often stressed that stability and reusability of photocatalysts are factors of key importance for their practical applicability [38]. To investigate the reusability of bismuth molybdate coating BMO3 (the most efficient one of the array), the photodegradation experiments for methylene blue and rhodamine B were repeated five times using the same sample. The repeatability results are illustrated in Figure 8. As can be seen, there was no significant loss of photocatalytic activity after 5 repeat cycles for either of the two dyes used. This confirms that the as-prepared bismuth molybdate coating exhibited satisfactory reusability after 5 cycles during the dye photodegradation tests. Additionally, XRD analysis of the samples was carried out after the reusability experiments; the XRD patterns indicated that there were no changes in the crystal structure of the photocatalytic coatings after 5 cycles of photodegradation of either dye.

## 4. Conclusions

The present work demonstrates that bismuth molybdates can be deposited by reactive magnetron co-sputtering from two metallic targets. All the coatings were characterized with relatively low band gap values (2.9–2.6 eV). The bismuth:molybdenum ratio of the coatings can be varied by varying the power applied to the targets. Despite the fact that increasing relative bismuth content helped to reduce the band gap of the coatings, the best results were obtained for the coating with a Bi:Mo ratio of approximately 2:1, which corresponds to stoichiometric Bi_2_MoO_6_. This is in a good agreement with information on magnetron sputtered bismuth tungstate coatings published earlier [24], where the highest photocatalytic efficiency was achieved for a coating with a Bi:W ratio of 2:1. The crystallinity of the bismuth molybdate coatings after annealing at 673 K was confirmed by Raman spectroscopy data, and the crystal phases were identified by XRD. The data on photocatalytic efficacy of the bismuth molybdate coatings against two different dyes showed similar trends with the coating with the optimum Bi:Mo ratio (64:36 at.%, respectively) was found to be the most efficient photocatalyst under visible light irradiation against both methylene blue and rhodamine B dyes. Reusability of the coatings for dye degradation showed that there was no significant decrease of photocatalytic activity after 5 cycles of MB or RhB photodegradation.

Overall, bismuth molybdate coatings demonstrated good potential as visible light-driven photocatalytic materials that can be applied for the photodegradation of organic pollutants. The combination of a relatively low crystallization temperature and low band gap value makes bismuth molybdate an interesting candidate for photocatalytic applications. Optimization of deposition conditions and surface properties of the bismuth molybdate coatings, as well as evaluation of their photocatalytic efficacy against different pollutants than dyes, will be investigated in the follow-up work.

## Abbreviations

The following abbreviations are used in this manuscript:

XRD	X-ray diffraction
XPS	X-ray photoelectron spectroscopy
SEM	scanning electron microscopy
EDX	energy dispersive spectroscopy
MB	methylene blue
RhB	rhodamine B
UV	ultraviolet
